# An Efficient Algorithm for Sensitively Detecting Circular RNA from RNA-seq Data

**DOI:** 10.3390/ijms19102897

**Published:** 2018-09-24

**Authors:** Xuanping Zhang, Yidan Wang, Zhongmeng Zhao, Jiayin Wang

**Affiliations:** 1 School of Electronic and Information Engineering, Xi’an Jiaotong University, Xi’an 710049, China; zxp@mail.xjtu.edu.cn (X.Z.); wyd199502@stu.xjtu.edu.cn (Y.W.); zmzhao@mail.xjtu.edu.cn (Z.Z.); 2 Shaanxi Engineering Research Center of Medical and Health Big Data, Xi’an Jiaotong University, Xi’an 710049, China

**Keywords:** RNA-seq, *de novo* detection, local similar sequence, high sensitivity

## Abstract

Circular RNA (circRNA) is an important member of non-coding RNA family. Numerous computational methods for detecting circRNAs from RNA-seq data have been developed in the past few years, but there are dramatic differences among the algorithms regarding the balancing of the sensitivity and precision of the detection and filtering strategies. To further improve the sensitivity, while maintaining an acceptable precision of circRNA detection, a novel and efficient *de novo* detection algorithm, CIRCPlus, is proposed in this paper. CIRCPlus accurately locates circRNA candidates by identifying a set of back-spliced junction reads by comparing the local similar sequence of each pair of spanning junction reads. This strategy, thus, utilizes the important information provided by unbalanced spanning reads, which facilitates the detection especially when the expression levels of circRNA are unapparent. The performance of CIRCPlus was tested and compared to the existing *de novo* methods on the real datasets as well as a series of simulation datasets with different configurations. The experiment results demonstrated that the sensitivities of CIRCPlus were able to reach 90% in common simulation settings, while CIRCPlus held balanced sensitivity and reliability on the real datasets according to an objective assessment criteria based on RNase R-treated samples. The software tool is available for academic uses only.

## 1. Introduction

Circular RNA (circRNA) is a class of important members of non-coding RNA family [1], which is considered as abundant [2], stable [3], and ubiquitous in diverse eukaryotic organisms [4,5] and may present a high degree of conservatism across evolutions [3,6]. CircRNAs are those RNA molecules in which an upstream 5′ splice site covalently linked a downstream 3′ splice site as a circle (named as back-splicing) [1,7], and the closed-loop structure outperforms linear structure in resistant to RNA digestion [4]. CircRNAs usually lack the poly(A) tails and reveal complicated structures which contains one or multiple exons, as well as introns, intergenic, and UTR regions [7,8,9]. Benefiting from RNA-Seq technology, more and more circRNAs have been identified and validated, and accumulated studies reported and emphasized the importance of them in many physiological processes [1,4,5], and associated to various complex diseases, e.g., digestive system cancers [10], breast cancer [11], diabetes [12], etc. Some circRNAs were observed to be functional, such as suppressing the activity of specific microRNAs [13], upregulating/downregulating the expressions of specific genes [14]. Recently, a number of studies have shown the potential of circRNAs on multiple translation processes, which implies that some circRNAs may be translated to peptides or proteins in vivo [15,16]. Further explorations on circRNAs are strongly suggested, which will be of far-reaching significance especially on disease occurrence, development, and precision diagnosis and treatments [17,18,19].

Detecting circRNAs is a basic, but crucial step for any related investigations [20,21]. In the experimental researches, RNase R is the popular protocol to enrich circRNAs [22]. Along with the popularity of RNA-seq, it becomes the major way of identifying circRNAs from RNA-seq data by computational methods [1,20,21,23]. Many state-of-the-art algorithms have been proposed for circRNA detection, while several important comparison studies carefully discussed the advantages and disadvantages among different algorithmic strategies [20,21]. According to the requirement of gene annotation information, the existing approaches, varying in detecting and filtering methods, are classified as *de novo* detection and annotation-dependent detection [20,21]. In addition, some data processing pipelines further introduce combined strategies to enhance the performance of circRNA detection [23,24]. In general, the annotation-dependent methods, such as CIRCexplorer [25] or MapSplice [26] are often more reliable in detecting the circRNAs that consist of annotated fragments but limit the usages on the species without complete annotation data. It also may suffer an accuracy loss when the circular junctions deviate the boundaries of exons. On the contrary, find_circ [5], CIRI [27], CIRI2 [28] and other *de novo* methods are able to handle more complicated cases [7,8,9], where one of the computational challenges is balancing the sensitivity and precision of detecting and filtering the candidates [21].

According to the current strategies on circRNA detecting and filtering, improving the accuracy of a detection algorithm is still an urgent need [20,21]. On the algorithmic level, the existing approaches could be improved in several aspects. Here we use CIRI2 as an example because it is suggested as one of the reputable algorithms and often outperforms in multiple comparison tests [21,27,28]. CIRI2 first utilizes a paired chiastic clipping (PCC) signal filter based on the local re-alignment results provided by BWA–MEM. Then, it establishes a probabilistic model to estimate the possible origins of a set of sequential segments (*k*-mers), each of which may have multiple mapping positions. This model not only improves the accuracy of detection but enhances the sensitivity as well. However, the PCC signals are often unreliable, either missed or incorrect, when the local regions for re-alignment involve complex junctions [27,28]. The flaws may originate from the following aspects: (1) BWA–MEM always selects the optimal alignment results in local re-alignment process, where some segments and their alignments may be ignored due to unbalanced segmentation. For example, it is possible that a back-spliced junction (BSJ) read has a much shorter segment flanking the junction comparing to the other one. However, these segments usually either cannot be mapped to the reference genome or are discarded by the mapper to avoid multiple “false-positive” alignments, both of which lead to the lack of PCC signals; (2) The shorter the segment that the algorithm cuts from a read, a higher the mapping error will be. The fortuitous matching may obscure the junction boundary between two separately mapped segments. In this case, the mapper often strapped in a deviation on compact idiosyncratic gapped alignment report (CIGAR) value. Thus, the insufficient PCC or CIGAR signals activate the filters or cross-validation filter, both of which lead to the lack of PCC signals as well; (3) Although the algorithm scans the unbalanced junction reads for the second round after clustering the circRNA candidates, the BSJ reads are not always able to provide paired signals due to the commonly low expression levels of circRNAs compared to the linear transcriptomes which consists of similar exons and/or introns, which present both 5′ splice site and 3′ splice site at the same time. This disables the algorithm and causes it to inadequately capture all of the BSJ reads. 

From the above discussion, better incorporating the imperfect BSJ reads would intuitionally enhance the local data signals and further improve the performance of circRNA detection. Thus, in this article, we proposed an efficient algorithm for circRNA detection, implemented as CIRCPlus. CIRCPlus is a *de novo* detection approach, which does not rely on the annotation information. CIRCPlus is supposed to be given a set of pair-ended reads with mapping results, which can be easily obtained by mapping tools, e.g., BWA–MEM, Bowtie2 [29]. CIRCPlus incorporates the local multi-alignments (fragment similarities) between two sets of the BSJ reads spanning a candidate circular junction, which directly overcomes the dependence on PCC signals. Benefiting from this, the algorithm is further able to unbiasedly identify more subtypes of circRNAs, including (1) small circRNA, whose length is around or shorter than one insert-size; (2) complex circRNAs, which consists of complex circular sequences, e.g., involving one or multiple intron-retained fragments; and (3) a circRNA, which harbors one or more short exons around the junction. We conducted multiple groups of experiments to compare the proposed algorithm to the existing approaches when the simulation configurations alter. The experimental results demonstrate that CIRCPlus outperforms other *de novo* approaches on both sensitivity and *F* measurement in most of the cases, while CIRCPlus maintains a high level of precision as well. In addition, CIRCPlus held balanced sensitivity and reliability on the read datasets according to an objective assessment criteria based on RNase R-treated samples.

## 2. Results and Discussion

To test the detection performance of CIRCPlus, we conducted the experiments on a series of simulation datasets with different configurations, and compared the performance of CIRCPlus to three popular *de novo* methods, which are CIRI [27], CIRI2 [28], and find_circ [5]. We also compared the outputs of CIRCPlus and evaluated the circular nature of each predicted species by its resistance to RNase R treatment known to specifically enrich for circRNA [7]. In the following experiments, two important metrics, sensitivity and precision, were calculated to show the performance of the methods. The sensitivity and precision are defined as:

(1) Sensitivity=TP/(TP+FN)

(2) Precision=TP/(TP+FP)

where TP is the true positive; FP is the false positive; and FN is the false negative. To evaluate the performance on balancing sensitivity and precision, *F*1-score was also employed, which is calculated by the following formula:

(3) $$F1-score=\frac{2\times Sensitivity\times Precision}{Sensitivity+Precision}$$

The greater the *F*1-score, the better the comprehensive detection performance. 

### 2.1. Generating the Simulation Datasets

CIRI-simulator [27] is a specific simulation tool for non-canonical transcripts. Here, we used CIRI-simulator to generate simulated reads and evaluate the performance of CIRCPlus, CIRI, CIRI2, and find_circ. CIRI-simulator requires two input files: a FASTA formatted reference sequence and a GTF or GFF formatted annotation file. A list of simulated circRNAs and FASTQ formatted files are then generated. The list is the true set for performance evaluation, while FASTQ formatted files are the inputs of the detection methods. Notably, parameters, such as read length, read depth (for both circRNAs and linear RNAs), sequencing error rate, and insert size can be customized by users. The parameter settings of the methods were listed in Supplementary Table S1.

To test the performance of CIRCPlus, we first generated the simulation datasets under different configurations. We selected the read lengths of 40, 50, 60, 80, 100, 125 and 150 bp, and altered the average read depths of 3-, 5-, 10-, 20-, 30-, 50-, and 70-fold to simulate sequencing reads, respectively. Read amounts were determined by the sequencing coverage and read length in each dataset. To avoid the alignment errors across multiple chromosomes, we applied the whole hg19 genome as the reference for alignment, but simply used chromosome 1 (length of 249250621) to generate simulated sequencing data. In detail, chromosome 1 from hg19 full set and its GTF annotation file (Gencode version 18) downloaded from [30] were used as the reference and annotation, respectively. For each dataset, the outputs were compared to the list to calculate the sensitivity and precision using custom scripts. We evaluated the performance from the following several aspects.

### 2.2. Analysis of Detection Sensitivity

#### 2.2.1. Detection Sensitivity under Different Read Depths of CircRNAs

We first focused on how the expression levels of circRNAs affect the performance of detection methods. Paired-end reads were generated from the reference genome with an increasing in the average read depths of circular transcripts, which alter from 3-, 5-, 10-, 20-, to 50-fold, while the average read depths of linear transcripts was kept on 10- fold. For each dataset, we calculated the sensitivity of CIRCPlus, CIRI, CIRI2, and find_circ.

**Figure 1:**
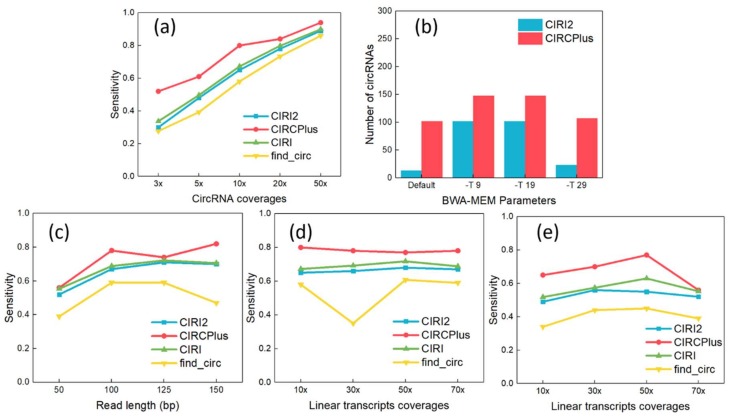
(**a**) Sensitivity analyses under different read depths of circRNAs (The read length was fixed to 100 bp). (**b**) Different parameter configurations of BWA–MEM affected the performance of detection on true positives (TP). (**c**) Sensitivity analyses under different read lengths. (**d**) Sensitivity analyses under different read depths of linear transcripts (The read length was fixed to 100 bp). (**e**) Sensitivity Analyses under different read depths of linear transcripts (The read length was fixed to 50 bp).

As shown in Figure 1a, the sensitivity of circRNA detection rose steadily, where CIRCPlus maintained 80% higher sensitivities when the read depths was equal or greater than 10-fold. The sensitivity reached as high as 94% at 50-fold read depth. Notably, even at the extremely low read depth of 3-fold, sensitivity was still kept more than 50% by CIRCPlus. In the comparisons, CIRI and CIRI2 reflected lower sensitivities varying from 30% at 3-fold to 89% at 50-fold, which were always less than CIRCPlus. Thus, CIRCPlus can hold higher sensitivities under different read depths comparing to CIRI, CIRI2, and find_circ.

#### 2.2.2. Detection Sensitivity under Different Configurations of BWA–MEM

CIRCPlus and other methods require aligned reads as input, which are normally generated by BWA–MEM. However, different parameter settings of BWA–MEM may lead to the different performance of CIRCPlus and CIRI2. According to CIRI2, it suggests alternative settings for parameter -T of BWA–MEM, which enables the alignments with low mapping scores. It was reported to improve the performance when the read length was short. Thus, we simulated short reads with 60 bp read length and tested the performance under different configurations of BWA–MEM. Read mapping was performed by using default parameters except “-T INT”. The function of -T is to filter out the alignments whose score is less than INT from the output. In the following experiments, the parameters were set to default, -T 9, -T 19, and -T 29, respectively. The results were shown in Figure 1b, and we observed that CIRCPlus always had higher TPs than CIRI2 did, and presented robust when the parameter altered (from 102 to 148). On the other hand, the results of CIRI2 varied more widely (from 13 to 102), and exposed little sensitive when the parameter was set to too low or too high. According to these results, we may say that CIRCPlus holds higher sensitivity and more robust on detection under different mapping configurations, while the CIRI2 also show great performance under suitable parameters. In the following experiments, we do not consider the read length shorter than 40 bp because CIRI2 cannot well handle the dataset whose read length is less than 40 bp. To conduct fair comparisons, we adopted the suggested parameter (-T 19) by CIRI2 when the read length was less than 60 bp.

#### 2.2.3. Detection Sensitivity under Different Read Lengths

We also tested the performance of CIRCPlus on variable read lengths. In this group of experiments, the read lengths varied from 50 to 150 bp, while the average read depth of circRNAs was set to 10-fold and the average read depth of linear transcripts was set to 70-fold. The results are shown in Figure 1c. From Figure 1c, although the detection sensitivities of different methods were gradually increasing along with the increasing of read length, it was obvious that CIRCPlus had higher detection sensitivity than CIRI, CIRI2, and find_circ for most of the cases. Specifically, when the read length was set to 100 bp, which is the most popular length, CIRCPlus identified more than 90% of the pre-set circRNAs, while CIRI and CIRI2 only reached around 67%. This means that CIRCPlus is efficient for different read lengths, and achieve a stably high sensitivity compared to CIRI2 (65% versus 51%) and other methods.

#### 2.2.4. Detection Sensitivity under Different Read Depths of Linear Transcripts

Some detection methods often misclassify the BSJ reads when the read depths of circRNAs are lower than the read depths of linear transcripts [20,21]. The reason is that low expressed circRNAs may have few supporting junction reads, which increases the difficulty of identifying the BSJ reads from the mass “noisy” reads supporting linear transcripts. In this group of experiments, the average read depth of circRNAs kept at 10-fold, while the average read depths of linear transcripts varied ranging from 10- to 70-fold. Two different read lengths are considered, which are 100 and 50 bp, respectively. For read length of 100 bp, the experiment results were shown in Figure 1d, where CIRCPlus always had a higher sensitivity than others. The sensitivity of CIRCPlus remained stably around 80% when the average read depths of linear transcripts varied ranging from 10- to 70-fold, while those of other were around 60–65%. When the read length trimmed to 50 bp (used “-T 19” parameter for BWA-MEM alignment), the results were shown in Figure 1e. In contrast to Figure 1d, the sensitivities had decreased generally. However, CIRCPlus still reached above 60% for most of the cases, while that of the find_circ decreased to less than 40%. Therefore, CIRCPlus is also suggested to have little higher sensitivity no matter the circRNAs read depths are interfered by the data noises from the linear transcripts with similar segments.

### 2.3. Analysis of Comprehensive Performance of Detection

To evaluate the comprehensive performance, detection precisions under different read depths of circRNAs were also tested in the experiments. We set the average read depth of linear transcripts to 10-fold and generated the paired-end reads from the reference genome with the average read depths of circular transcripts of 5-, 10-, 20-, and 50-fold, respectively. The detection precisions of CIRCPlus were calculated and compared to those of others under different read depths of circular transcripts, as shown in Table 1. We found that the precisions of CIRCPlus were a little lower than those of CIRI2 and find_circ, both of which maintained among the highest precisions under different read depths of circular transcripts from 5- to 50-fold. The precisions of CIRCPlus and CIRI were almost same. However, although CIRCPlus presented lower precisions than CIRI2 and find_circ did, such precisions were acceptable and able to provide meaningful results for most of the downstream analyses because of high sensitivity.

**Figure 2:**
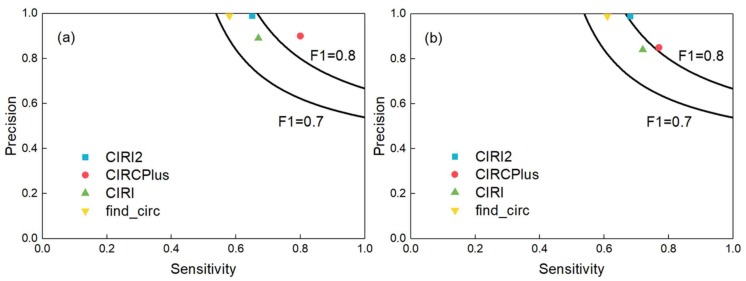
Comparisons of CIRCPlus and three popular circRNA detection methods. (**a**) Sensitivity and precision comparison among CIRCPlus and other methods, when the average read depth of linear transcripts was set to 10-fold (read length was set to 100 bp). (**b**) Sensitivity and precision comparison among CIRCPlus and other methods, when the average read depth of linear transcripts was set to 50-fold (read length was set to 100 bp)

Normally, not only high precision but also high sensitivity is required for circRNA detection. A good algorithm should balance the sensitivity and precision [20]. Thus, we computed the *F*1-score to measure the comprehensive performance of detection. In this group of experiments, we set the average read depth of circular transcripts to 10-fold, and generated the paired-end reads with read lengths of 40, 50, 60, 80, 100, 125 and 150 bp, respectively, where for each read length configuration, the read depths of linear transcripts altered among 10-, 30-, 50-, and 70-fold, respectively. The full experiment results were listed in Supplementary Table S2. Figure 2 showed the *F*1-scores of the four methods when the read length was 100 bp and the average read depths of linear transcripts were 10-fold (a) and 50-fold (b), respectively. According to Figure 2, CIRCPlus had the highest *F*1-scores in both datasets under different read depths across all four methods. After applied different simulation configurations on these methods, we may say that CIRCPlus often has a more comprehensive performance advantage than CIRI, CIRI2, and find_circ, especially when the reads are trimmed, or the read depths of linear transcripts are close to the read depths of circular transcripts. Notably, when the read length was shorten to 40 bp, about 40% circRNAs were detected by CIRCPlus, whereas none of them were detected by CIRI or CIRI2 under default parameters of BWA-MEM. Even used “-T 19” parameter for BWA-MEM, CIRCPlus still registered more true circRNAs than CIRI and CIRI2 (60% versus 30%). In addition, the *F*1-scores of CIRCPlus were much higher than others in most of the cases (Supplementary Table S2). Therefore, we could conclude that CIRCPlus performs better in balancing the sensitivity and precision under different configurations, and thus it is suggested to have better comprehensive performance on circRNA detection.

### 2.4. Benchmarking CircRNA Detection Using CIRCPlus

The RNase R enrichment dataset is a gold standard for benchmarking circRNA detection methods. Here, a pair of datasets of HEK293 cell line [31], one of which sequenced the sample with RNase R treatment (accession number SRR3479244), while the other sequenced the sample without RNase R treatment (accession number SRR3479243), were used for evaluating the detection performance of CIRCPlus. The datasets were downloaded from the SRA database. We adopted these datasets because they were also used to compare the performance of detection methods in previous studies [28]. We used the following criteria, proposed in [28], to evaluate candidate circRNAs passed basic filter (BSJ read count ≥4 in the dataset without RNase R treatment): If a candidate circRNA detected by CIRI2 or CIRCPlus is obviously enriched after RNase R treatment (at least 3-fold increase on BSJ read count), then it is labeled as a true positive; Otherwise, if the candidate circRNA is not detected or is detected but supporting by few BSJ reads after RNase R treatment, then it is labeled as a false positive. The ratio of false positives across all of the predictions is then calculated as false discovery rate (FDR) of the method.

**Figure 3:**
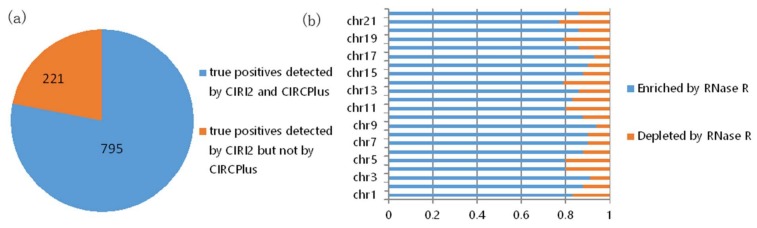
(**a**) Overlap of circRNAs predicted by CIRCPlus and CIRI2 on the HEK293 datasets. (**b**) RNase R resistance of each chromosome predictions only detected by CIRCPlus.

In the consideration of no validation for the true positives detected by computational methods from the 150 bp HEK293 datasets, we further explored the overlap of the predicted circRNAs by CIRCPlus and CIRI2 on the HEK293 datasets. To quantify the performance of CIRCPlus, we compared the true positives that predicted by CIRCPlus under the criteria described above to those detected by CIRI2. The results were shown in Figure 3. From Figure 3a, the predictions of CIRI2 and CIRCPlus on the same datasets showed a significant overlap. 795 out of 1016 candidate circRNAs reported by CIRI2 were also reported by CIRCPlus. To further evaluate the precision, after filtered the candidate circRNAs by CIRCPlus default constraints, which required 1) at least two reads supporting each splice site, and 2) the supporting BSJ reads had reliable mapping scores, there were exclusively 855 candidates predicted by CIRCPlus in the final output. We found 732 true positives out of 855 candidates based on the BSJ read enrichment by incorporating the dataset treated with RNase R. The results, chromosome by chromosome, were shown in Figure 3b (sex chromosomes were not considered). Taken together, the above performance evaluations demonstrated that CIRCPlus not only detected the vast majority of true positives which were detected by other popular method, CIRI2, but also reported the candidates with a quite low percentage (14%) of false positives.

## 3. Materials and Methods

### 3.1. Framework of the Proposed Approach

It is necessary to identify circRNAs at a high sensitivity with high precision regardless of circRNA expression levels and read alignment results. By analyzing the BSJ reads within a circRNA, we find that every BSJ read that spans the back-spliced site is comprised by two terminal sequences flanking the junctions. It is obvious that any two of BSJ reads must have local similar sequences. Here, we propose a novel algorithm, CIRCPlus, based on local sequence alignment between any two of BSJ reads and combined with a systematic filtering strategy to remove false positives, multithreading is implementation in CIRCplus to facilitate large dataset analysis. In CIRCPlus, the Sequence Alignment/Map (SAM) alignment file is generated by the BWA–MEM (the mapping tool for RNA-seq data which supports split mapping and calculates the junction information) and then used for identifying BSJ reads. The SAM file gives all alignment records of each read. CIRCPlus extracts all unmapped reads from the SAM file as the potential BSJ reads. These unmapped reads can be classified into two clusters according to alignment results from the SAM file. One cluster, called left-junction-cluster, contains the unmapped reads where the unmatched parts are on the left side, while the other, called right-junction-cluster, contains the unmapped reads where the unmatched parts are on the right side. Since a candidate BSJ read is considered to indicate a circRNA only when the mapping position of its paired read locates in the putative circRNA region on the reference genome, the unmapped reads in the two clusters can be filtered based on PEM (Paired-End Mapping) signals to refine the candidate BSJ reads. To identify the putative BSJ reads, considering that any pair of BSJ reads mapped in the same circular junction should both have a coverage region flanking 5′ and 3′ splice sites, and have an overlapped region termed as “local similar sequence”, the candidate BSJ reads are further filtered based on local similar sequences to obtain the putative BSJ reads. Here, a dynamic programming algorithm is employed to determine whether two reads from two clusters have local similar sequence, and outputs local similar sequence of these two reads. Finally, all of the putative BSJ reads are clustered to make the putative BSJ reads from the same circRNA in the same class.

The process of CIRCPlus can be summarized as following four steps:

(1) Extracting and classifying the unmapped reads;
(2) Filtering the unmapped reads based on PEM signal to get the candidate BSJ reads;
(3) Identifying the putative BSJ reads based on local similar sequences;
(4) Clustering all putative BSJ reads from the same circRNA.

**Figure 4:**
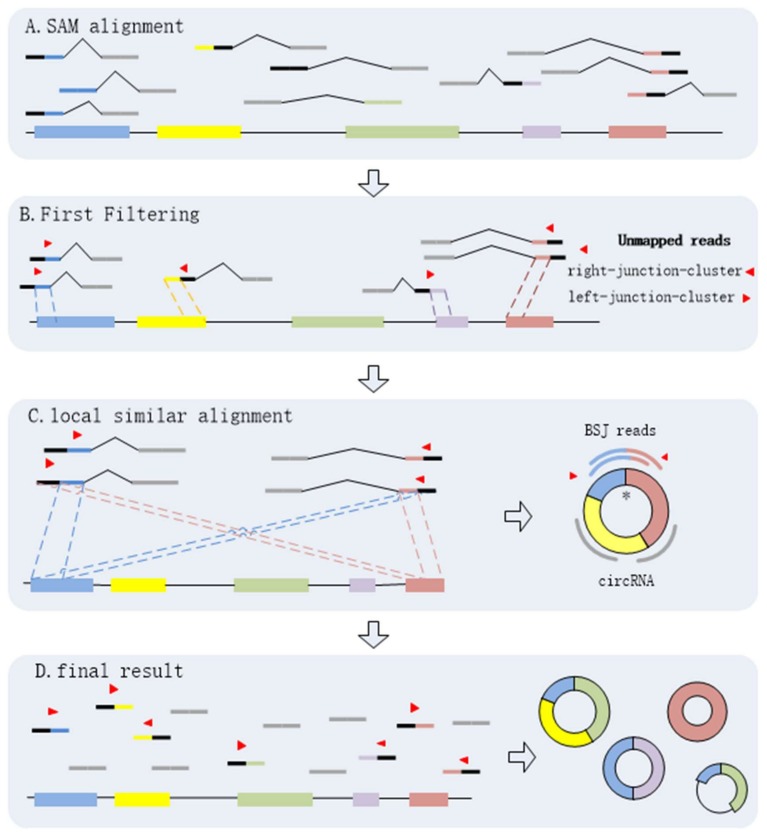
CIRCplus workflow. (**A**) Extracting and classifying the unmapped reads from the alignments of each pair of reads. (**B**) Filtering the unmapped reads based on PEM signal to get the candidate BSJ reads. (**C**) Identifying the putative BSJ reads based on local similar sequences. (*) denotes the circular junction. (**D**) Clustering all putative BSJ reads from the same circRNA. In each box, the longest line on the bottom denotes the reference genome, where the colored regions represent different exons, and the black lines between exons simply represent introns. The short lines denote reads. For each read, the red triangle represents its direction, and a pair of dotted lines indicate a possible alignment of it mapping to the reference genome.

Through the above steps, CIRCPlus is implemented to identify and characterize circRNAs. After the first and second steps, the candidate BSJ reads including the junction reads with PCC signals as well as unbalanced junction reads are obtained. Unlike the existing methods, e.g., CIRI, which extract a fixed size of anchors from the unmapped reads to identify potential backspliced junctions, CIRCPlus ignores such limitations for detecting unbalanced junction reads, and thus more candidate junction reads can be obtained for the next step. In the third step, CIRCPlus utilizes the local alignment to obtain the whole BSJ reads whether it has PCC signals or not, and the local alignment is useful to different read length during detection. As a result, the sensitivity of detection has been improved. The overall workflow of CIRCplus is shown in Figure 4, each step will be described in detail separately.

### 3.2. Extracting and Classifying Unmapped Reads

**Figure 5:**
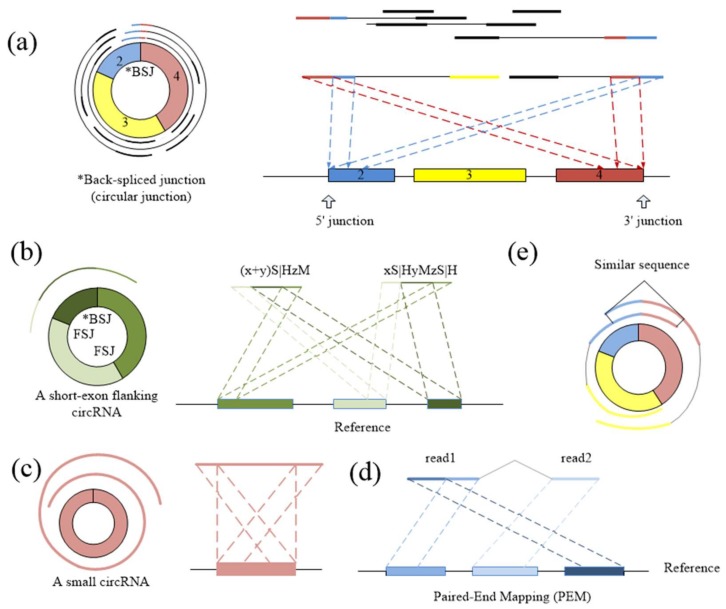
(**a**) The typical two-segment junction reads align to the reference genome separately in reverse orientation. (**b**) If one exon flanking the junction is shorter than the read length, the rest of the segment can be aligned to the nearby exon(s) contained in the circRNA. (**c**) If the length of a circRNA is shorter than the read length, it may map to the reference genome in another three-segment style. (**d**) The pair read of a junction read should align within the inferred circRNA area. (**e**) Any two of BSJ reads within a circRNA should have a similar sequence. The dotted lines suggest the possible alignment of each part of BSJ reads.

CIRCPlus requires the SAM alignment file generated by BWA–MEM. BWA–MEM aligns reads to the reference genome by mapping approaches and outputs to the confident alignment results to SAM file. During read mapping, some BSJ reads are mapped in 5′ splice site while some are mapped in 3′ splice site. Notably, some BSJ reads will have a pair of above alignment results, which named “paired chiastic clipping signals” (PCC signals) in CIRI. A typical junction is separately mapped with the reference in a corresponding two-segment style, as shown in Figure 5a. Here, CIRCPlus extracts the unmapped reads which have corresponding alignment results and classifies the reads with CIGAR value in the form of xS|HyM in left-junction cluster (suppose that locate at 5′ splice site), while the reads with CIGAR value in the form of xMyS|H are put in right-junction cluster (suppose that locate at 3′ splice site).

In addition to the typical junction reads which are mapped to the reference genome in a two-segment style, some circRNAs have complex alignment features. In one case, if the exon flanking the junction of a circRNA is shorter than the read length, then some junction reads of the circRNA may inconsecutively map to the reference in a three-segment style, where two segments map to two exons flanking the junction and the third segment maps to the proximal part of the exon adjacent to the short flanking exon contained in the circRNA (Figure 5b). In another case, if a circRNA is smaller than the read length, then it may also align to the reference in another form of the three-segment style, where two terminal segments separately overlap the terminal parts of the area where the middle segment aligns (Figure 5c). In both situations, CIGAR values present the alignment features of xS|HyMzS|H at 5′ splice site and 3′splice site, and those reads are then put into the left-junction cluster and right-junction cluster simultaneously.

For each paired-end read, two ends are considered respectively. That is, each end is clustered into left-junction cluster or right-junction cluster according to its corresponding alignment records. When the reads have multiple mapping, the corresponding alignment records are separately taken into account, because sequencing errors or fortuitously matching bases may obscure the junction boundary between two separately aligned segments. Therefore, this method considers not only the PCC signals and unbalanced junction reads, but also the alignment results which just mapped at one of the junction sites. It simplifies the complexity of the second scanning of SAM in the existing method, and the candidate set can be expanded no matter whether it outputs the suitable alignment results or not. Here, the unmapped reads are put into two clusters based on CIGAR values, where left-junction cluster represents the potential chimeric junction reads which may be located at 5′ splice site within a circRNA, while right-junction cluster represents the potential chimeric junction reads which may be located at 3′ splice site.

### 3.3. Filtering Unmapped Reads

Because the two segments of a bona fide junction read represent the boundary of a circular RNA, the candidate junction read is considered to indicate a circRNA only when its paired read mapping position is within the putative circRNA region on the reference genome (Figure 5d). Therefore, the unmapped reads in left-junction or right-junction cluster can be further filtered based on PEM signal to remove false positives.

For the junction reads in the left-junction cluster, the mapping positions represent the possible left boundaries of the circRNA, so the paired read should fall within the downstream area (5’ to 3’ direction) of its junction reads in the reference genome. Furthermore, for the reads in right-junction cluster, the mapping positions represent the possible right boundaries of the circRNA, so the paired read should fall within the upstream area (3’ to 5’ direction) of the junction reads. Here, the read direction is used to determine whether it is supported by the PEM signals. On one hand, the junction reads in the left-junction cluster should be the positive direction, while the paired read should be the negative direction. On the other hand, the junction reads in the right-junction cluster should be the negative direction, while the paired reads should be the positive direction. As a consequence, the paired-end reads with the same direction are filtered.

### 3.4. Identifying Putative BSJ Reads

**Figure 6:**
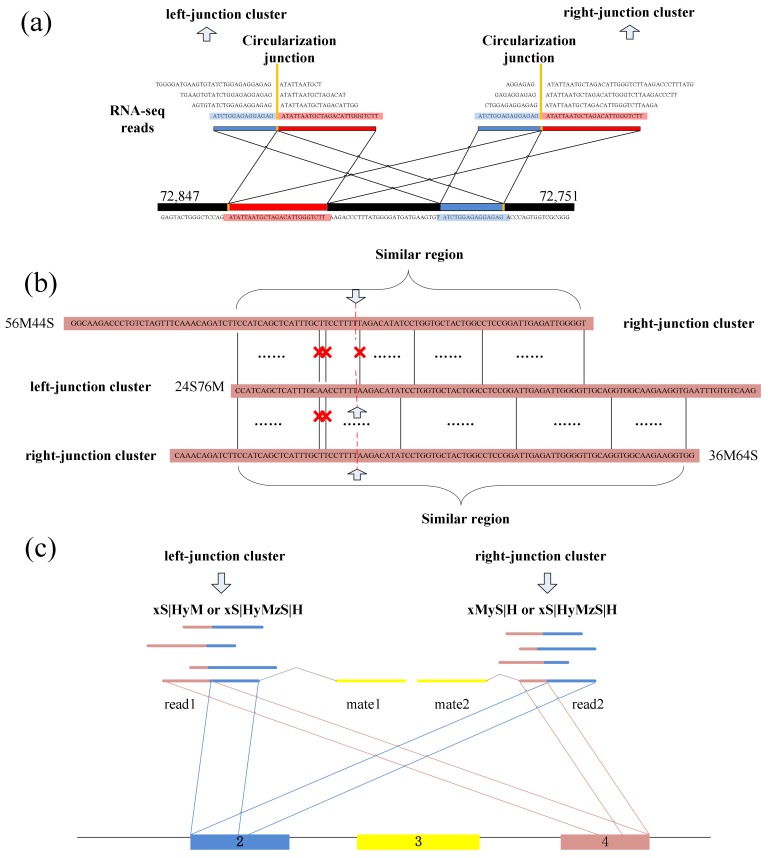
(**a**) A read from left-junction cluster should have a similar sequence with a read from right-junction cluster. (**b**) Two reads from two clusters are accepted during the detection. A red cross denotes a mismatch base-pair in the similar region. (**c**) Clustering the BSJ reads within a circRNA.

Spanning the circular junction reads, termed as “BSJ reads”, are spliced by sequences flanking the junction. Those reads have different segment lengths flanking 5′ splice site and 3′ splice site respectively. As a consequence, a similarity sequence must exist between any two of BSJ reads within a circRNA, as shown in Figure 5e. Therefore, a pair candidate BSJ reads mapped in the same circular junction should both have a coverage region flanking 5′ and 3′ splice sites. Local alignment, for determining similar regions between two reads, can be used to judge whether it outputs the similar sequence or not and then check the similar sequences if it spans the circular junction, as shown in Figure 6a.

In the local alignment, the two reads for alignment should satisfy the following restrictions:

(1) The read from left-junction cluster should be located at the upstream of the read from right-junction cluster, and mapping distance between them along the genome reference should be reasonable.
(2) According to the PEM signals, their paired reads should fall within the region indicated by both junction reads.
(3) The two reads should be aligned to the same chromosome.
(4) The two reads should have the reasonable mapping scores.

For each pair of reads satisfying the above requirements, these two reads are compared to obtain their similar sequence. If a pair of reads exists similar sequences across the circular junction, these two reads are further detected. Here we suggest to use multithreading to analyze all alignment results between any supported pair of junction reads. 

A dynamic programming algorithm is implemented to find the optimal local alignment with respect to the scoring system (which includes the substitution matrix and the gap-scoring scheme). Because the sequencing errors may lead to mismatch in the local sequence alignment between two BSJ reads, the number of mismatches is uncertain. So, a threshold for the numbers of mismatches and dashes is preset to filter the similar sequences, which makes the results better for detection. The threshold can be adjusted by user, as different read length may use different mismatch thresholds to obtain better precision. Figure 6b shows an acceptable example during the detection.

### 3.5. Clustering All Putative BSJ Reads Within A CircRNA

Numerous of pairs of BSJ reads now have been generated though the previous steps, and for any pair, its results contain a read mapped to 5′ splice site and a read mapped to 3′ splice site within a circRNA. These BSJ reads are clustered subsequently according to their junction loci. Though fortuitously matching bases may obscure the junction boundary, the nearby junction loci can also be employed to cluster all circRNA junction reads, as shown in Figure 6c.

Due to the split alignment strategy of BWA–MEM, the short splice read may contain false positive BSJ reads. To prevent false predictions resulting from fortuitously alignment results, we consider the mapping quality and the number of supporting BSJ reads to filter the false positive. After investigation of all reads, CIRCPlus summaries the mapping positions of all detected candidate BSJ reads. By comparing the read counts and the mapping quality of the reads, CIRCPlus further determines whether these reads reliably reflect a circRNA junction and whether the candidate circRNA should be kept until the final output or not.

## 4. Conclusions

In this article, we focused on the computational problem that identifying circRNAs from the next-generation RNA sequencing data. It is suggested that circRNAs with high abundance may associate with important functions. Detecting circRNAs directly from sequencing data is a computational challenge in bioinformatics. Several state-of-the-art methods are proposed and able to handle circRNA detections with different advantages and disadvantages, specifically on balancing the sensitivity and precision. We proposed a novel algorithm, CIRCPlus, to detect circRNAs from RNA-seq data. The proposed method directly computes the alignment results provided by BWA–MEM and implements an efficient *de novo* algorithm which simplifies the twice scanning strategy. CIRCPlus identifies the BSJ reads based on the local similar sequences, which was not considered in any of the existing methods. This strategy is able to identify more supporting BSJ reads of the circRNAs, where are usually ignored or misclassified by the existing methods. Benefiting from this, CIRCPlus is able to report circRNAs with high sensitivity and acceptable precision, and further obtain higher *F*1-score at the same time. Because the step “local alignment between two reads” is unaffected by read length, CIRCPlus also shows the better performance than the existing *de novo* methods on trimmed reads. The experimental results demonstrate that CIRCPlus is quite robust on both sensitivities and precisions varying the read depths, read lengths, the numbers of the preset circRNAs, as well as the different parameter settings on BWA-MEM, comparing to three popular methods CIRI2, CIRI, and find_circ in most of the simulation configurations. It is also tested on the paired HEK293 datasets, where CIRCPlus outperformed on both high sensitivity and low FDR. According to our limited knowledge on circRNA detection methods, we believe that CIRCPlus provides an efficient and unbiased circRNA detection tool for future circRNA studies. In the next study, we will further concentrate on increasing the precision in our algorithm.

## Figures and Tables

**Table 1:** Precision analysis of detection under different read depths of circular transcripts.

Read Depth	5-fold	10-fold	20-fold	50-fold
CIRCPlus	0.80	0.90	0.88	0.93
CIRI	0.90	0.89	0.91	0.91
CIRI2	0.99	0.99	0.98	0.98
find_circ	0.99	0.99	0.99	0.99

## Supplementary Materials

Supplementary materials can be found at http://www.mdpi.com/1422-0067/19/10/2897/s1.

## Abbreviations

CircRNA	Circular RNA
PEM	Paired-End Mapping
BSJ	Back-spliced Junction
FSJ	Forward-spliced Junction
