# Supplementary material for: An Efficient Algorithm for Sensitively Detecting Circular RNA from RNA-seq Data
Source: Int J Mol Sci. 2018 Sep 24;19(10):2897. doi: 10.3390/ijms19102897 (PMC6213952; doi:10.3390/ijms19102897)
Supplement: Supplementary file 1 [file ijms-19-02897-s001.zip › Supplement Table S1-revised.pdf]

Supplementary Table S1.

Tool	*De novo*	Mapper	Dependencies	Language	Version
CIRCplus	Yes	BWA-MEM	None	Perl Java	N/A
CIRI	Yes	BWA-MEM	None	Perl	1.0.2
CIRI2	Yes	BWA-MEM	None	Perl	2.0.3
find_circ	Yes	Bowtie2	Pysam samtools Bedtools	Python	1.2
