# Supplementary material for: An Efficient Algorithm for Sensitively Detecting Circular RNA from RNA-seq Data
Source: Int J Mol Sci. 2018 Sep 24;19(10):2897. doi: 10.3390/ijms19102897 (PMC6213952; doi:10.3390/ijms19102897)
Supplement: Supplementary file 1 [file ijms-19-02897-s001.zip › Supplement Table S2-revised.pdf]

Supplementary Table S2.

read_length	linear_transcript_ coverage	BWA-MEM '-T'	find_circ			CIRI			CIRI2			CIRCplus		
			sensitivity	precision	F1-score	sensitivity	precision	F1-score	sensitivity	precision	F1-score	sensitivity	precision	F1-score
40	10	default	0.11	0.99	0.198	0	0	0	0	0	0	0.4	0.77	0.5264957
		-T19				0.36	0.89	0.5089809	0.33	0.98	0.4937405	0.58	0.83	0.6828369
	30	default	0.12	0.99	0.2140541	0	0	0	0	0	0	0.4	0.52	0.4521739
		-T19				0.35	0.89	0.5068391	0.32	0.98	0.4824615	0.55	0.53	0.5398148
	50	default	0.11	0.98	0.1977982	0	0	0	0	0	0	0.37	0.50	0.4252874
		-T19				0.31	0.76	0.4422517	0.28	0.98	0.4355556	0.56	0.42	0.48
	70	default	0.13	0.99	0.2298214	0	0	0	0	0	0	0.42	0.37	0.3934177
		-T19				0.3	0.78	0.4333333	0.29	0.98	0.4475591	0.58	0.47	0.5192381
50	10	default	0.34	0.99	0.5061654	0	0	0	0	0	0	0.60	0.90	0.72
		-T19				0.52	0.93	0.6657122	0.49	0.98	0.6533333	0.65	0.85	0.7366667
	30	default	0.44	0.99	0.6092308	0	0	0	0	0	0	0.67	0.82	0.7374497
		-T19				0.57	0.84	0.6820325	0.56	0.98	0.7127273	0.70	0.88	0.7797468
	50	default	0.45	0.99	0.61875	0	0	0	0	0	0	0.67	0.80	0.7292517
		-T19				0.63	0.78	0.696325	0.55	0.99	0.7071429	0.77	0.80	0.7847134
	70	default	0.39	0.99	0.5595652	0	0	0	0	0	0	0.48	0.58	0.525283
		-T19				0.55	0.80	0.6543967	0.52	0.98	0.6794667	0.56	0.54	0.5498182
60	10	default	0.50	0.99	0.6644295	0.15	0.90	0.2585278	0.13	0.98	0.2295495	0.73	0.93	0.8179518
	30	default	0.52	0.99	0.6818543	0.12	0.75	0.2051282	0.12	0.98	0.2138182	0.72	0.82	0.7667532
	50	default	0.51	0.99	0.6732	0.12	0.78	0.2113821	0.12	0.98	0.2138182	0.69	0.77	0.7278082
	70	default	0.47	0.99	0.6373973	0.14	0.60	0.2327837	0.14	0.98	0.245	0.69	0.67	0.6798529
80	10	default	0.61	0.99	0.754875	0.55	0.90	0.6808607	0.53	0.98	0.687947	0.77	0.92	0.8383432
	30	default	0.60	0.99	0.7471698	0.63	0.84	0.7193303	0.57	0.98	0.7207742	0.75	0.85	0.796875
	50	default	0.58	0.99	0.731465	0.68	0.88	0.7688675	0.59	0.98	0.7365605	0.80	0.85	0.8242424
	70	default	0.56	0.99	0.7153548	0.60	0.84	0.7033201	0.54	0.98	0.6963158	0.77	0.81	0.7894937
100	10	default	0.58	0.99	0.731465	0.67	0.89	0.7660561	0.65	0.99	0.7847561	0.80	0.90	0.8470588
	30	default	0.35	0.98	0.5157895	0.69	0.85	0.7629375	0.66	0.99	0.792	0.78	0.83	0.8042236
	50	default	0.61	0.99	0.7529643	0.72	0.84	0.7739515	0.68	0.99	0.8062275	0.77	0.85	0.8080247
	70	default	0.59	0.99	0.7393671	0.69	0.83	0.7524394	0.67	0.98	0.7958788	0.78	0.82	0.7995
125	10	default	0.54	0.99	0.6988235	0.69	0.95	0.7968127	0.75	0.98	0.849711	0.91	0.88	0.8947486
	30	default	0.60	0.99	0.7471698	0.75	0.92	0.8274583	0.69	0.98	0.8098204	0.79	0.77	0.7798718
	50	default	0.52	0.99	0.6818543	0.70	0.87	0.7735615	0.68	0.98	0.8028916	0.80	0.80	0.8
	70	default	0.59	0.99	0.7393671	0.72	0.89	0.7969264	0.71	0.98	0.823432	0.74	0.73	0.734966
150	10	default	0.46	0.98	0.6261111	0.71	0.95	0.8104628	0.68	0.98	0.8028916	0.85	0.84	0.8449704
	30	default	0.51	0.99	0.6732	0.70	0.88	0.781315	0.72	0.99	0.8336842	0.82	0.81	0.8149693
	50	default	0.46	0.99	0.6281379	0.73	0.88	0.8006499	0.70	0.98	0.8166667	0.79	0.80	0.7949686
	70	default	0.47	0.99	0.6373973	0.71	0.90	0.7910777	0.70	0.98	0.8166667	0.82	0.74	0.7779487
